# Induction of Genomic Instability in a Primary Human Fibroblast Cell Line Following Low-Dose Alpha-Particle Exposure and the Potential Role of Exosomes

**DOI:** 10.3390/biology10010011

**Published:** 2020-12-28

**Authors:** Eman Mohammed Elbakrawy, Ammar Mayah, Mark A. Hill, Munira Kadhim

**Affiliations:** 1 Department of Biological and Medical Sciences, Faculty of Health and Life Sciences, Oxford Brookes University, Oxford OX3 0BP, UK; emanoxford2018@gmail.com (E.M.E.); mayahahj@gmail.com (A.M.); 2 Department of Radiation Physics, National Center for Radiation Research and Technology, Atomic Energy Authority, 3 Ahmed El-Zomor Al Manteqah Ath Thamenah, Nasr City, Cairo 11787, Egypt; 3 Gray Laboratories, MRC Oxford Institute for Radiation Oncology, University of Oxford, ORCRB Roosevelt Drive, Oxford OX3 7DQ, UK; mark.hill@oncology.ox.ac.uk

**Keywords:** genomic instability, bystander, exosomes, alpha-particles, low dose

## Abstract

**Simple Summary:**

Exposure to the naturally occurring radioactive gas Radon, and the resulting alpha-particle emitting progeny, dominates human exposure and is the second largest cause of lung cancer after smoking. The work presented shows that not only can very low doses of alpha-particles produce DNA damage in normal HF19 fibroblast cells (detected using comet and micronuclei assays) in the irradiated cell population, but also results in an enhanced yield of DNA damage in the progeny of these cells out to 10 and even 20 population doublings. This persistent elevation of DNA damage is consistent with genomic instability, a well-recognised feature of many tumours with its ability to generate genetic diversity in the dividing population. Environmental exposure is normally associated with a small fraction of cells being irradiated with a single alpha-particle, while the vast majority of cells remain unirradiated. Here, we demonstrate an enhanced yield of DNA damage observed at 10 and 20 population doublings even when less than 1% of the cell population is traversed, with a response similar to that observed when essentially all cells are irradiated. The finding also highlights the potential of exosomes produced by irradiated cells contributing to DNA damage observed in unirradiated bystander cells.

**Abstract:**

Purpose: To study the induction of genomic instability (GI) in the progeny of cell populations irradiated with low doses of alpha-particles and the potential role of exosome-encapsulated bystander signalling. Methods: The induction of GI in HF19 normal fibroblast cells was assessed by determining the formation of micronuclei (MN) in binucleate cells along with using the alkaline comet assay to assess DNA damage. Results: Low dose alpha-particle exposure (0.0001–1 Gy) was observed to produce a significant induction of micronuclei and DNA damage shortly after irradiation (assays performed at 5 and 1 h post exposure, respectively). This damage was not only still evident and statistically significant in all irradiated groups after 10 population doublings, but similar trends were observed after 20 population doublings. Exosomes from irradiated cells were also observed to enhance the level of DNA damage in non-irradiated bystander cells at early times. Conclusion: very low doses of alpha-particles are capable of inducing GI in the progeny of irradiated cells even at doses where <1% of the cells are traversed, where the level of response was similar to that observed at doses where 100% of the cells were traversed. This may have important implications with respect to the evaluation of cancer risk associated with very low-dose alpha-particle exposure and deviation from a linear dose response.

## 1. Introduction

Exposure to the naturally occurring radioactive gas radon and its resulting alpha-particle emitting progeny dominates human exposure to radiation and is now known to be the second largest cause of lung cancer after smoking [1]. As exposure is primarily via inhalation, the largest dose of alpha-particles is received by lung cells, although other organs may also receive a significant dose [2]. Exposure to alpha-particles may also result from their use in targeted radiotherapy [3] as well as occupational exposures (e.g., in the nuclear industry) [4].

The properties of alpha-particle irradiation and associated damage differ significantly from conventional low-LET (linear energy transfer) radiation, such as X-ray and gamma-ray. While low LET radiation produces predominantly simple DNA double-strand breaks (DSB) which are repaired with a half-time of ~20 min, approximately, 90% of DSB produced by alpha-particles are complex DSB (DSB with additional strand breaks and/or base damage within 10 base pairs) [5,6] with a corresponding decrease in repair rate and corresponding probability of faithful repair [7]. Additionally, the passage of an alpha-particle through a nucleus will result in a highly heterogeneous pattern of damage through a cell producing closely spaced correlated DNA breaks along the narrow path of the particle. The close proximity of these correlated breaks along the track of the α-particle also result in an enhanced probability of illegitimate re-joining, which can result in gene mutations and complex chromosomal rearrangements [8,9]. At low doses to a tissue or cell population, many cells will not be traversed by an alpha-particle, but those that are traversed will receive a significant dose with multiple sites of damage [9]. The response of cells to low fluence or single alpha-particle traversals is important as this corresponds to typical human exposures; for example, typical environmental indoor radon exposures correspond to 0.002–0.009 alpha-particle traversals per year [10].

In addition to conventional radiation-induced effects (chromosome damage, mutation and cell death), ionising radiation (IR) can also induce genomic instability (GI), which is defined as an increased rate of accumulation of genomic alterations, which may appear at delayed time-points in the progeny of the irradiated cells. These alterations include ongoing new changes in chromosomes, gene mutations and enhanced cell death [11]. GI is also known to be a prominent enabling characteristic of cancer [12]. Interestingly, GI is not only observed in the progeny of irradiated cells, but also in the progeny of non-irradiated bystander cells as a result of cell-cell communication with alpha-particle irradiated cells [13,14]. Rather than a single pathway, it is likely that the bystander response is multifaceted with a range of signalling pathways involved [15], showing a range of intercellular signalling. The influence of cell signalling following irradiation, however, is not just restricted to nearby cells, but has been observed at distant sites in the body; these are commonly referred to as abscopal effects [16]. Previous studies have demonstrated on a range of cell lines that alpha-particles are able to induce genomic instability in the progeny or irradiated and unirradiated bystander cells. These include murine haemopoietic cells derived from irradiated stem cells [14,17], human bone marrow cells [18] and human lymphocytes [13], with similar effects observed by other groups as discussed in various reviews [11,19]. However, none of these studies have previously investigated how the induction of genomic instability in the progeny of an irradiated cell population varies with dose down to environmentally relevant levels.

Exosomes are membrane-bound extracellular vesicles (50–150 nm) which are released by cells into the extracellular environment [20]. These are known to have an important role in the intercellular communication, such as tissue repair and the immune system, influencing cell phenotype as well as the nervous system [21]. The released exosomes interact with target cells through expressed growth factors, bioactive lipids and membrane receptors, which could cause a direct stimulation for the target cells [22]. Kumar Jella et al., [23] 2014, suggested that exosomes may cause epigenetic changes in the recipient cells by transmit of genetic information through shuttling mRNA and microRNA. The study showed that exosomes form irradiated cells could also mediate the bystander effects in human keratinocyte cells (HaCaT cells) [23]. Kadhim et al. [24] (2018) have reported that exosomes mediated signal transfer from irradiated (low LET) cells to bystander cells and induced GI. While in a number of areas, particularly neurodegenerative disease and cancer pathology, exosome research field is well established [25], this is not currently the case in the radiobiology field, especially in connection with high LET radiation. A better understanding of the role of exosome bystander in radiation-induced alterations is clearly needed, given their potential role in genomic instability, radiation carcinogenesis and radiotherapy.

The goal of the current study was to explore the role of low doses of alpha-particles (0.0001 Gy to 1 Gy) on the induction of GI in normal human diploid lung fibroblast cells. Micronuclei (MN) and comet assays were used as a marker of GI either in the irradiated cell population following exposure or after approximately 10 and 20 population doublings. In this study we also tested the hypothesis that exosomes from the irradiated cells could induce DNA damage in nonirradiated bystander cells. 

## 2. Materials and Methods

### 2.1. Cell Culture

The cells used in this study are primary non-transformed human lung fibroblasts. These cells were derived from non-transformed human fibroblasts from the lung of a 14-week-old female foetus, designated HF19 by Cox and Masson [26] from MRC, Harwell Campus, Oxfordshire, UK [26]. These cells were cultured in Minimum Essential Medium with Earle’s salts without L-Glutamine, and supplemented with 10% Foetal bovine serum (Sigma-Aldrich, Gillingham, UK: F7524); 1% L-Glutamine (Gibco, Loughborough, UK: 25030); 1% non-essential amino acids (Gibco: 11140) and 1% (*v*/*v*) penicillin/streptomycin solution (Sigma: P0781) in a humidified 5% CO_2_ incubator at 37 °C. Cells were grown initially in 25 cm^2^ (T25) and later in 75 cm^2^ (T75) tissue culture flasks for several population doublings during cell propagation. Cells were passaged twice weekly.

### 2.2. Exosome Isolation 

Cell media (supernatant) were collected from cells 24 h after irradiation. Cell media were centrifuged at 2000× *g* to remove dead cells and apoptotic bodies and any other larger contaminants, as described by Al-mayah et al. [27,28]. The supernatant was then centrifuged at 10,000× *g* for 30 min to clear the sample of microvesicles. This followed by collecting the supernatant and ultracentrifuged at 120,000× *g* for 1 and half hours. The supernatant was discarded and exosome pellet collected in 200 µL of two times 0.22 µm-filtered Phosphate buffered saline (PBS).

### 2.3. Exosome Characterisation

Exosome characterisation for concentration and size was performed on Izons qnano transient resistive pulse sensing platform, as described by Al-mayah et al. [29]. Briefly, exosomes were collected in 200 µL of twice filtered PBS. Samples were diluted 1:20 in twice filtered PBS. Samples were run through disposable Nano-pores for a maximum of 10 min or until 500 exosomes have been counted.

### 2.4. Exosome Transfer for Bystander Experiments (Exosome Bystander)

Exosomes (from each irradiated group and controls) were isolated and collected in 200 µL PBS; 10 µL was removed and kept aside for quantification (Size and concentration). The remaining exosome sample was added to HF19 un-irradiated cells and incubated for 24 h before subjecting to viability (see Supplementary Data Figures S1 and S2) and comet assays.

### 2.5. Irradiation

#### 2.5.1. Alpha-Particle Irradiation

Due to the short range of alpha-particle tracks, cells must be irradiated as a monolayer. In brief, the cells were seeded at a density of 2 × 10^5^ in 2 mL of media into each of the irradiation dishes (20 Hostaphan dishes per group). These dishes consisted of Pyrex glass cylinders (30 mm internal diameter, Chance Glass Ltd., Malvern, UK) with bases of 2.5 µm Hostaphan film on which the cells attached and grow [30]. The cells were then incubated undisrupted for ≈44 h at 37 °C, 5% CO_2_ before irradiation to allow them to form a sub-confluent monolayer of cells. Just prior to irradiation, cell thickness in spare dishes was assessed by confocal microscopy to ensure that they had an average thickness <10 µm. All irradiations were performed using the University of Oxford ^238^Pu alpha-particle source [31]. The cells were irradiated with alpha-particles with incident energy of 3.26 MeV (LET ≈ 121 keV/µm) with up to 10 dishes are mounted in a wheel at 3.0 revs·min^−1^ above a sector plate defining the edges of the beam incident on the dish (ensuring all positions on the dish were exposed for an identical time). The dose rate was varied by changing the size of the sector plate along with the size of an aperture directly above the source to deliver a range of doses (0, 0.0001, 0.001, 0.01, 0.1, 0.5 and 1 Gy) with all the exposures delivered in less than 2 min. A sham irradiated group (Control) was set up in parallel. The cells were incubated (37 °C, 5% CO_2_) for 1 h following irradiation and then collected from the Hostaphan-based dishes by trypsinisation. A fraction of cells (2 × 10^4^) was subjected to the comet assay which was utilised to measure total DNA damage in all experimental groups, whilst other fractions (1.5 × 10^6^) were seeded into 1xT75 flasks with their irradiated medium (15 mL) for the micronucleus assay. The remainder was then further propagated for assessment of intermediate (10 population doublings or 5 passages) and delayed effects (20 population doublings or 10 passages). The irradiated cells were seeded in irradiated media for 24 h (80–90% confluence) and then subcultured with fresh media (new nutrition) until reaching 10 and 20 population doublings following irradiation.

#### 2.5.2. X-ray

Cell irradiations were performed at the Gray Institute for Radiation Oncology & Biology, Department of Oncology, University of Oxford, utilising the MXR321 X-ray machine operating (Comet, Flamatt, Switzerland) at 250 kV constant potential and 12 mA for irradiation with a high dose rate, and 250 kV and 0.3 mA with a low dose rate. The X-ray energy deposition is sparse and uniform, ensuring that each cell in the ionisation track path receives some irradiation even at low doses.

Cells were seeded at 3 × 10^6^ in T175 tissue culture flasks and incubated for ≈24 h at 37 °C, 5% CO_2_ before irradiation. Cells were then exposed to 1.5 Gy X-ray (iso-effective killing dose for 0.4 Gy alpha-particles, ~30% Survival) and the cells were subjected to comet assay 48 h following irradiation.

### 2.6. Measurements of Nuclear and Cellular Area

For a given dose, the number of alpha-particle tracks traversing the cell or nucleus is dependent on its area, and at low doses not all cells are necessarily traversed [32]. Thus, measurements of the cellular and nuclear area were made to calculate the average number of alpha-particle traversals per cell and per nucleus, by different doses.

**Figure 1:**
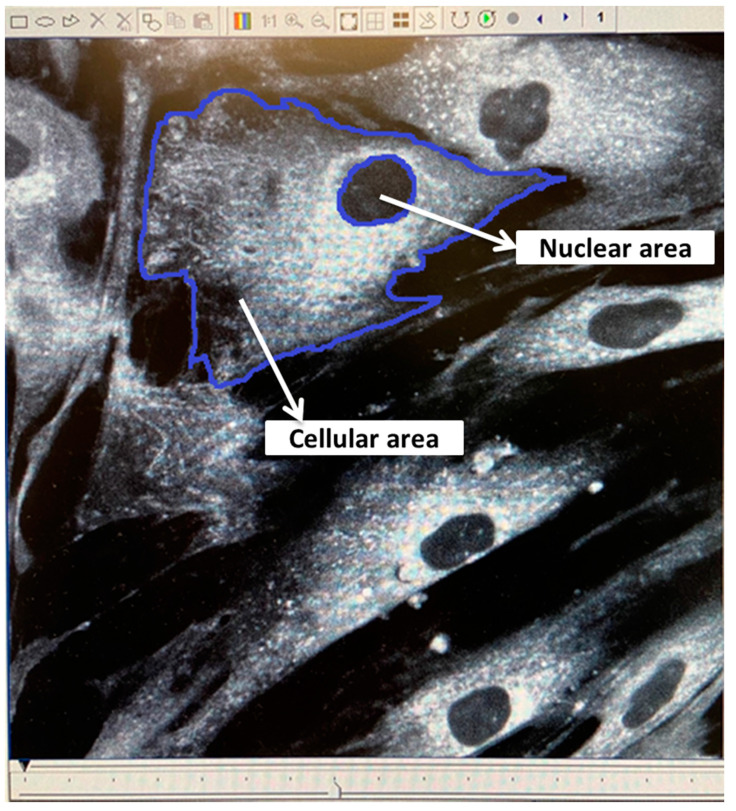
Confocal images of living HF19 cells stained with DIOC6. The image was taken by a confocal laser scanning microscope using a ×60 oil lens. The dimension of the resulting image is 196 µm × 196 µm.

Immediately prior to irradiation, two randomly chosen spare Hostaphan-based dishes of HF19 cells were stained with DIOC6 (3, 3′-dihexyloxacarbocyanine iodide, Sigma), a fluorescent dye which stains the cell mitochondria, endoplasmic reticulum and vesicle membranes. Random ‘saved’ multiple cell images were taken of horizontal sections across these stained monolayer cells. The images were taken by a confocal laser scanning microscope (BioRad-Lasersharp 2000 confocal microscope coupled to a Nikon TE2000 microscope with an ion argon laser operating at 488 nm wavelength). This allowed subsequent measurement of the living cells’ nuclear area and cellular area as shown in Figure 1. The computer programme ‘Image J’ was initially used to manually draw around the circumference of each nucleus and cell, by which the computer is able to calculate the area of each, as shown in Figure 1.

### 2.7. Micronucleus Assay

The frequency of micronucleus induction in HF19 cells was measured using the cytokinesis block protocol technique adapted from Erexson and colleagues [30,31]. Cells were collected from Hostaphan dishes at 1 h following irradiation and incubated for 4 h before adding 6 µg/mL cytochalasin-B and subsequently incubated for 40 h. Cells were then harvested, centrifuged at 200× *g* at RT (room temperature) for 10 min, the supernatant discarded and the remaining pellets were re-suspended. Once re-suspended, 1 mL of hypotonic solution (warmed KCl; 0.55 g potassium chloride (Sigma, P3911) and 100 mL ultrapure water kept in a 37 °C water bath) was subsequently added in a drop-wise manner followed by a further 10 mL KCl. The tubes were then incubated in a 37 °C for 5 min prior to adding three drops of 25% glacial acetic acid in methanol (3:1 fixative); all tubes were inverted once and centrifuged at 200× *g* for 10 min at RT. Following removal of the supernatant, the pellets were resuspended in 10 mL of 3:1 fixative (added drop-wise), and left at RT for 10 min. Cells were further centrifuged at 200× *g* for 10 min at RT; supernatant discarded and pellets re-suspended in 0.5–1 mL of 3:1 fixative depending on the pellet size. The resulting fixed cell suspension was dropped onto individually labelled, clean/degreased microscope slides and these were left to dry at RT before analysis. Additionally, micronucleus assay was used to investigate the induction of micronuclei in HF19 cells at 5 and 10 passages, approximately 10 and 20 population doublings, following irradiation.

### 2.8. Staining of Slides

**Figure 2:**
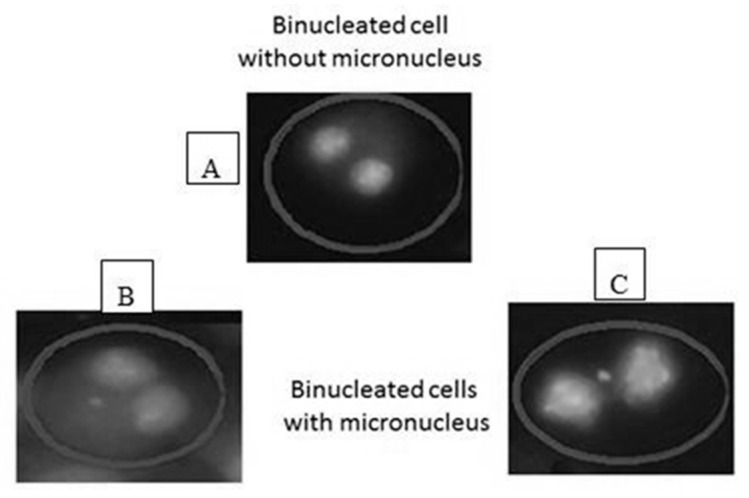
Scoring of binucleated cells: (**A**) a binucleated cell without micronucleus, (**B**,**C**) binucleated cells with micronucleus of different size. The cells were analyzed under ×40 magnification using fluorescent microscope.

Acridine Orange (AO) is a nucleic acid selective metachromatic red fluorescent stain, which is commonly used in cell cycle determination and enables visualisation of nuclear changes. A phosphate buffer was prepared using one tablet (pH 6.8) in one litre of distilled water (dH_2_O). In brief, two Coplin jars were each filled with 50 mL prepared buffer (pH 6.8); to the first jar 0.0031 g acridine orange (Sigma: A6014) was added. Slides were stained for 25 s in the acridine orange/buffer solution and then quickly dipped for a few seconds in the buffer-only jar [33,34]. Finally, they were left to dry at RT before analysis on a fluorescent microscope (Figure 2).

### 2.9. Scoring Micronuclei (MN)

Slides were coded and analysed in a blind and random fashion to avoid observer bias (i.e., slides were coded by a colleague in the research group). Micronuclei were scored only in binucleate cells (BN) and at least 500 binucleate cells were scored per group for four biological replicates. Micronucleus induction was quantified as the percentages of binucleate cells (BN) within micronuclei (%MN/BN) [35].

### 2.10. Alkaline Comet Assay (Single Cell Gel Electrophoresis)

The comet assay is a quick, sensitive and comparatively simple technique for the evaluation of DNA damage (predominantly detecting DNA single-strand breaks along with alkali-label sites) in individual cells [36]. The comet assay was carried out as explained by [37,38]. Microscope slides were plated with a thin layer of 1% normal melting point agarose solution (NMPA) (Sigma: A9539) by immersing the clean slides in agar. The excess was wiped from the back and the slides were laid to dry overnight and then kept in a microscope box. On the harvesting day, the cells were detached with 1.5 mL (0.025%) trypsin and a cell count was performed. For each group, 2 × 10^4^ cells were mixed with 200 L of 1% low melting point agarose (LMPA) (Fisher Scientific, Loughborough, UK: BP165). The NMPA pre-coated slides were laid on an ice-chilled metal plate and 200 µL of the LMPA cell suspension mixture was placed immediately onto the chilled slides. The cell-LMPA suspension was spread and flattened by putting a 24 × 50 mm glass coverslip on top. After 5–10 min, the coverslips were taken away to allow complete setting. The slides were then dipped in cold lysis buffer ((2.5 M NaCl, 100 mM Na2EDTA (pH 8.0), 10 mM Tris-HCl (pH 7.6), 1% Triton X-100 (pH > 10) and 1% DMSO). The lysis process was performed at 4 °C overnight in the dark. In a cold room (4 °C), the slides were laid horizontally in the alkaline electrophoresis buffer tank (0.3 M NaOH and 1 mM EDTA (pH 13)). The slides were then left for 30 min at 19 V and 200 mA. Finally, the slides were neutralised with neutralising buffer (0.5 M Tris-HCl (pH 7.5)) for 3 × 10 min then the slides were washed with dH_2_O to remove any remaining buffer. The slides were stained immediately with a 1:10,000 dilution of Diamond Nucleic Acid Dye (Promega, Southampton, UK: H1181) in the dark. The slides were left at 25 °C overnight and at least 500 cells per group were analysed using Komet 5.5 Image Analysis Software (Kinetic Imaging Technology/Andor, Berlin, Germany).

### 2.11. Statistical Analyses

During the scoring, the investigator was blinded to the sample treatment (e.g., non-irradiated/irradiated). The *p* values of raw data from all experimental groups were calculated to compare the treatment groups with its control. The Y error bars for all experimental groups were generated by calculating the standard error of the mean (±S.E.M.) for all groups. The Kolmogorov-Smirnov test was used to examine if the comet assay data were normally distributed. The test is sensitive to outliers. The comet assay data were not normally distributed. Therefore, data were subjected to a Mann-Whitney U test (SPSS statistics 21, Oxford Brookes University, Oxford, UK) to measure the *p*-value. As the results have extreme scores, the median was used instead of the mean. This is due to the insensitivity of the median to extreme scores where the mean is highly sensitive to them [39].

The MN data were shown not to have normal distribution; thus, it was further subjected to Fisher’s exact test to calculate the *p* values. *p* values ≤ 0.05 were considered statistically significant. The standard error of the mean (±S.E.M.) was calculated to generate error bars for all experimental groups.

## 3. Results

### 3.1. Calculating the Average Number of Alpha-Particle Track Traversals per Nucleus and per Cell

**Figure 3:**
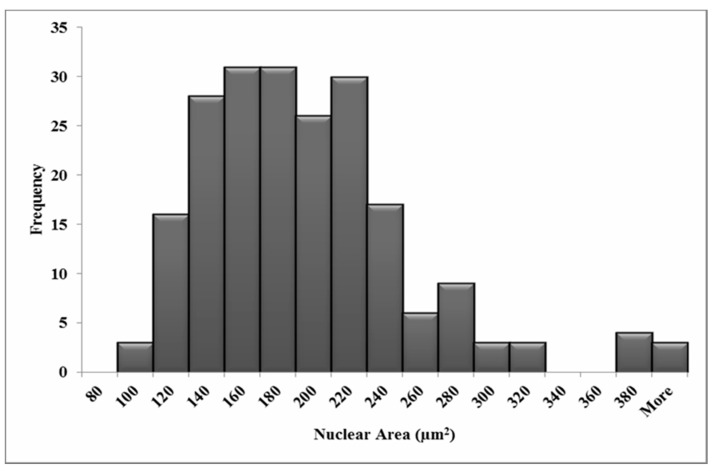
Measured nuclear area distributions for the HF19 cell line. The mean area is 189 µm^2^ for 211 cells over three experiments.

**Figure 4:**
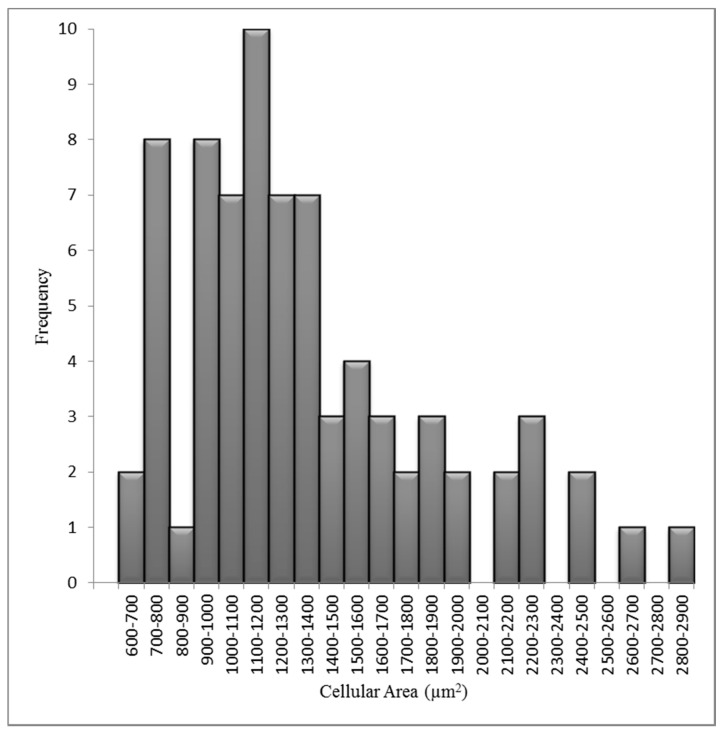
Measured cellular area distributions for the HF19 cell line. The mean cellular area is 1557 µm^2^ for 80 cells over three experiments.

The variation in nuclear and cellular area in the attached HF19 cells are shown in Figure 3 and Figure 4, respectively, and correspond to a mean nuclear area of 189 µm^2^ and cellular area of 1557 µm^2^. The average number of tracks (randomly distributed) per cell, N, traversing a mean nuclear area or cellular area, A (in µm^2^) for a given dose D (in Gy) is given by Equation (1).

N = AD/0.16 L(1)

where L is the LET in keV·µm^−1^, which is taken as 121 keV·µm^−1^ [32]. These tracks are following a Poisson distribution. Therefore, not all cells will be traversed by an equal number of alpha-particle tracks and at a given dose not all cells will be traversed. The fraction of cells traversed, F, by *n* tracks (*n* = 0, 1, 2, …) is given by Equation (2).

(2) $$F\;=\;\frac{N^{n}\;e^{-N}}{n!}$$

where N is the mean number of alpha-particles traversals per cell (Equation (2)).

The fractions of cells traversed by one or more tracks, F_1+_, is given by Equation (3):

F_1+_ = 1 − e^−*n*^ (3)

The resulting variation in the average number of traversals and percentage of cells/nuclei traversed as a function of doses used are show in Table 1. 

### 3.2. DNA Damage in HF19 Either Directly Irradiated or Bystander Cells Exposed to Exosomes from the Irradiated Cell Population

**Figure 5:**
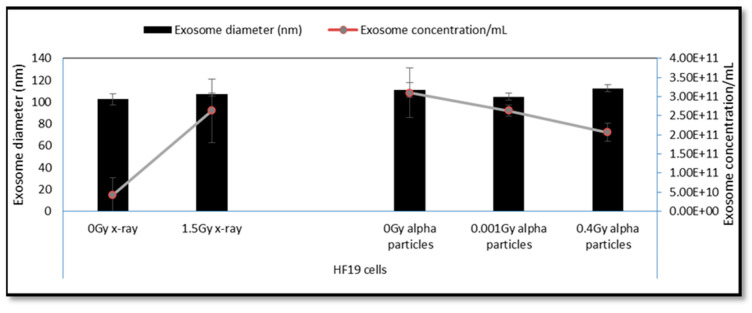
Exosome characterisation 24 h following direct X-ray and alpha-particle irradiation. A significant increase in exosome concentration was displayed by the 1.5 Gy X-ray-irradiated group compared to the corresponding control. However, a non-significant gradual decrease was observed by 0.001 Gy and 0.4 Gy alpha-particle-irradiated groups compared to the corresponding control. The data showed a non-significant change in the size of the exosomes for all irradiated groups compared to the corresponding controls.

**Figure 6:**
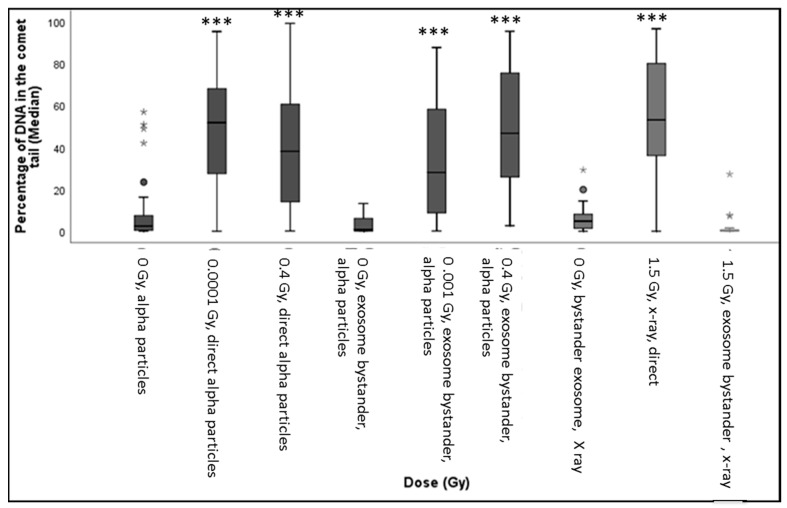
The box-plot shows the distribution of damage (including outliers showed as small circles in the figure) in HF19 cells after 48 h following completion of X-ray and alpha-particle irradiation. Specifically, it shows the percentage of DNA (damage) in the comet tail in HF19 cells 48 h following direct X-ray and alpha-particle irradiation and 24 h following exosome bystander transferred to non-irradiated cells. The DNA damage was measured using the comet assay (% tail DNA) (* = *p* ≤ 0.05, ** = *p* ≤ 0.01 *** = *p* ≤ 0.001).

The variation of exosome diameter and concentration released following exposure to X-rays (0, 1.5 Gy) and alpha-particles (0, 0.01, 0.4 Gy) is shown in Figure 5. While the size appears to be independent of treatment, a significant increase in exosome concentration was displayed by 1.5 Gy X-ray irradiated group compared to the corresponding control. Moreover, a gradual decrease in concentration was observed for the 0.001 Gy and 0.4 Gy alpha-particle irradiated groups compared to the corresponding control. The exosome characterisation experiment was repeated three times (three technical repeats). The difference in exosome expression between the “0 Gy X-ray” and the “0 Gy alpha-particles” samples is potentially due to the fact that the number of cells seeded in the 0 Gy X-ray samples and in the 0 Gy alpha-particles samples were not equal and were seeded in very different types of containers. Cells irradiated with 1.5 Gy X-ray along with 0.01 Gy and 0.4 Gy alpha-particles all demonstrated a significant increase in the DNA in comet tail (representative images (fluorescence microscopy) were included as Supplementary Data, Figure S3) assayed 48 h post exposure (Figure 6). A significant increase was also observed in the unirradiated bystander cells exposed to exosomes from both 0.001 Gy and 0.4 Gy alpha-particle irradiated cells but not cells treated with 1.5 Gy X-rays (Figure 6).

### 3.3. Early and Late DNA Damage in HF19 Cells Post Low and High Doses of Alpha-Particle Irradiation: Comet Assay

The alkaline comet assay was used to assess the total DNA damage in the HF19 cells caused by the various doses (0.0001–1 Gy) of alpha-particle irradiation at differing time points. The percentage of DNA in the tail, which represents the DNA damage, was scored in each group of approximately 1000 cells for two separate but parallel experiments performed at different times. 

**Figure 7:**
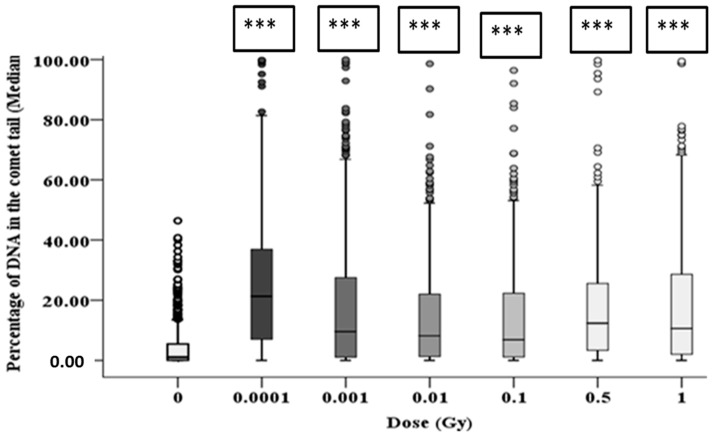
Percentage of DNA (damage) in the comet tail in HF19 cells 1 h following alpha-particle irradiation. HF19 cells were exposed to different doses of alpha irradiation and incubated for 1 h prior to performing the comet assay. The DNA damage was measured by the comet assay (% tail DNA) as combined data for 1000 cells which were scored from two separate, independent experiments. The box-plot shows the distribution of damage (*** = *p* ≤ 0.001).

**Figure 8:**
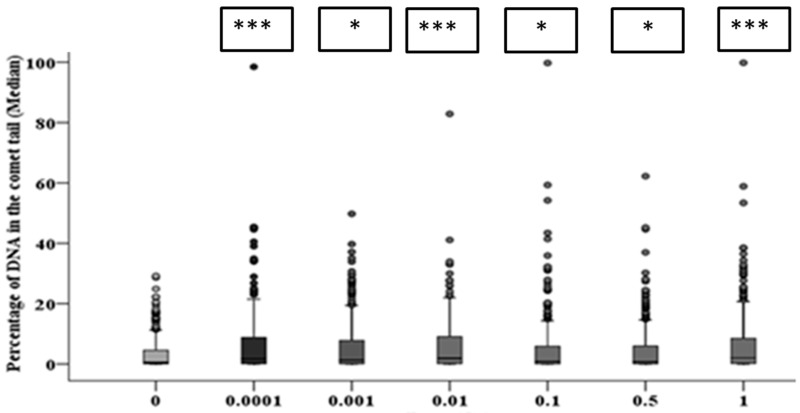
Intermediate responses within the progeny of alpha-particle irradiated cell populations after 10 population doublings following irradiation. Panel A shows the variation in median values as a function of dose for 1000 cells from both the two parallel but separate experiments. Panel B exhibits the DNA damage distribution of combined data from two parallel but separate experiments (* = *p* ≤ 0.05, *** = *p* ≤ 0.001).

**Figure 9:**
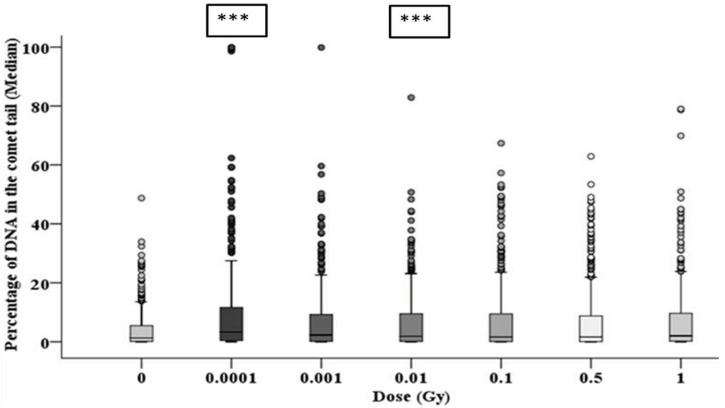
The delayed DNA damage in the progeny of alpha-particle irradiated cells following 20 population doublings determined using the comet assay. Panel A shows the variation in median values as a function of dose for 1000 cells from two parallel but separate experiments. Panel B exhibits the delayed DNA damage distribution in alpha-particle irradiated cells (*** = *p* ≤ 0.001).

The resulting induction of DNA damage after 1 h, 10 population doublings and 20 population doublings are shown in Figure 7, Figure 8 and Figure 9, respectively, with all control samples across the three time points (median values of 0.85 ± 0.48%, 0.47 ± 0.23% and 1.67 ± 0.27%, respectively) showing low levels of background DNA damage. In general, at 1 h following the alpha-particle exposure, an early large induction of DNA damage was observed across all irradiated groups compared to the corresponding control, as shown in Figure 7. Due to the nature of the alpha-particle tracks, the distribution of the damage is also presented using box plots, as it has been observed that not all nuclei displayed DNA damage after α-radiation, as some of the nuclei showed no tail formation (see Figure 7). All doses demonstrated a number of cells with tail DNA up to 50% and some cells irradiated with 0.0001, 0.001 and 1 Gy showed tail DNA up to 100%. Interestingly, the highest level of DNA damage was observed following an exposure of 0.0001 Gy, approximately 22-fold higher than the control.

After 10 population doublings, levels of DNA damage had dramatically reduced; however, they remained significantly above the corresponding control (Figure 8). The progeny of HF19 cells irradiated with 0.0001, 0.01 and 1 Gy alpha-particle radiation showed the highest level of total DNA damage 1.74 ± 0.40%, 1.83 ± 0.34% and 1.62 ± 0.37%, respectively, which was significantly higher (*p* ≤ 0.001) than the control, 0.47 ± 0.23%, as shown in Figure 8. There was a smaller, but still nearly twofold increase in DNA damage with the progeny of 0.001, 0.1 and 0.5 Gy cells, which was still significantly above control levels (*p* ≤ 0.05).

At 20 population doublings post irradiation, the level of total DNA damage was still relatively high and in line with the levels observed after 10 population doublings (and in some cases higher), as shown in Figure 9. However, at this later time point there was an observed increase in the level of damage observed in the controls (1.67 ± 0.27%); as a result, only the 0.0001 and 0.01 Gy groups showed significantly (*p* ≤ 0.001) higher induction of DNA damage. However, the distribution of damage was greater than that of control groups for all irradiated groups, suggesting an enhanced level of DNA damage-induced post radiation in the progeny of the original cell, even when the median level of damage was not significantly above the control (see Figure 9). 

### 3.4. Early and Late Induction of Micronuclei in Binucleate HF 19 Cells Post Low and High Doses of Alpha-Particle Irradiation: Micronucleus Assay

**Figure 10:**
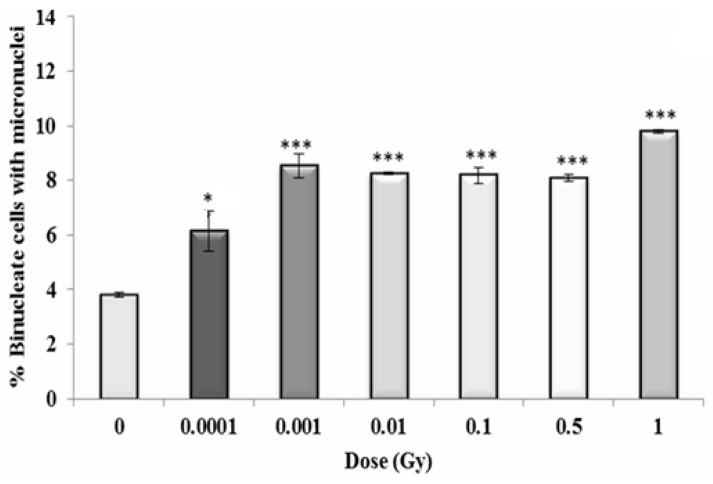
Percentage of binucleate cells containing micronuclei over total binucleate cells in control and irradiated HF19 cells at 5 h following irradiation. The figure represents the combined data for 1000 cells from two independent but parallel experiments as a function of dose. Error bars represent the standard error of the mean of replicate experiments (±S.E.M.) from the two independent experiments. *p*-values were calculated using Fisher’s exact test for comparison of data with associated un-irradiated control. * *p* ≤ 0.05, *** *p* ≤ 0.001.

**Figure 11:**
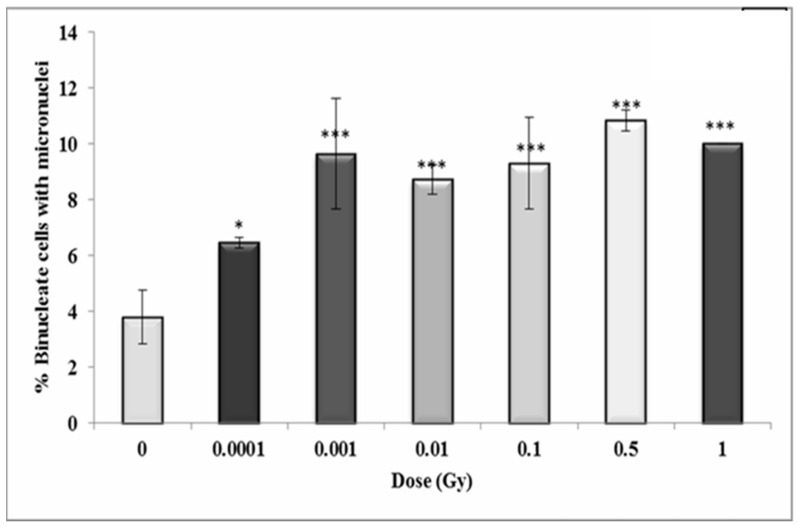
Percentage of binucleate cells containing micronuclei over total binucleate cells in control and irradiated HF19 cells at 10 population doublings. The figure shows an intermediate analysis of the variation in the percentage of binucleate cells containing micronuclei values as a function of dose for 1000 cells from two parallel but separate experiments. Error bars represent the standard error of the mean of replicate experiments (±S.E.M.). *p*-values were calculated using Fisher’s exact test. * *p* ≤ 0.05, *** *p* ≤ 0.001.

**Figure 12:**
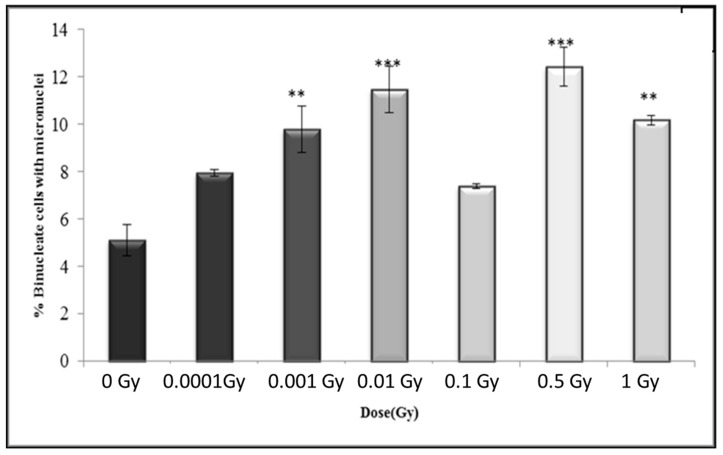
Percentage of binucleate cells containing micronuclei over total binucleate cells in control and irradiated HF19 cells at 20 population doublings following irradiation. The figure shows delayed analysis of the variation in the percentage of binucleate cells containing micronuclei as a function of dose for 1000 cells from two parallel but separate experiments. Error bars represent the standard error of the mean of replicate experiments (±S.E.M.). *p*-values were calculated using Fisher’s exact test. ** *p* ≤ 0.01, *** *p* ≤ 0.001.

The variation in micronucleus formation as a function of dose (0.0001 to 1 Gy) with the assay performed 5 h, 10 population doublings and 20 population doublings following irradiation are shown in Figure 10, Figure 11 and Figure 12, respectively. 

For the early response (5 h post irradiation), the data shows a 1.5-fold increase above control even for the lowest dose of 0.0001 Gy (*p* ≤ 0.05) with the response plateauing with an approximate twofold increase at 0.001 Gy and above (*p* ≤ 0.001), with a suggestion of a further increase (~2.5-fold) at the highest dose of 1 Gy (Figure 10).

In general, the results show a similar trend in the binucleate cells with micronuclei at 10 population doublings following irradiation as for the early time-point with an increase observed at 0.001 Gy (*p* ≤ 0.05), rising to a 2.5-fold increase at 0.001 Gy (*p* ≤ 0.001) with the response plateauing at higher doses (Figure 11). 

In general, the results after 20 population doublings indicating a similar enhancement above control following irradiation, but with a more variable response with increasing dose, with increase levels only statistically significant for 0.001 Gy, 0.01 Gy, 0.5 Gy and 1 Gy (Figure 12). The data also indicate a slightly higher response for the control (5.09 ± 0.22%) compared to control levels observed at 5 h (3.79 ± 0.19%) and 10 populations doublings (3.81 ± 0.19%).

The comet assay experiment was repeated four times (four technical repeat) and the micronucleus assay has the combined data for 1000 cells from two independent but parallel experiments as a function of dose.

Although 1000 cells were examined for comet and MN assays, the recommended sample size was calculated using G*Power since only two biological replicates were used. The G*Power results for the MN assay data showed that a sufficient number of binucleate cells was analysed in each irradiated group for a powerful statistical analysis except with the 0.0001 Gy group. Additionally, the same trend of sample size was observed with the comet assay results. However, the G*Power results for 0.1 and 0.01 Gy groups recommended repeating the comet assay at least one more time.

## 4. Discussion

The clonogenic survival probability of HF19 cells exposed to alpha-particles over the doses used for the alpha-particle exposures used (0.0001 Gy, 0.001 Gy, 0.01 Gy, 0.1 Gy, 0.5 Gy and 1 Gy) are 100%, 100%, 97%, 75%, 23% and 5%, respectively, based on a D0 of 0.34 Gy determined from pervious experiments using the irradiator [40]. The initial DNA damage induced by ionising radiation immediately following radiation is expected to increase linearly with the dose with an increasing number of tracks traversing the nucleus. It is, therefore, intriguing and counter-intuitive that a significantly raised level DNA damage was observed by the comet assay 1 h following irradiation for doses as low as 0.0001 Gy, with the response reducing slightly with increasing doses up to 0.1 Gy. While the response is more variable for a dose at 10 and 20 population doublings, at these later times a significant increase is observed at a dose of 0.0001 Gy. This non-linear dose response is also supported by the micronuclei data, where the early response (cytochalasin-B added 5 h post exposure) also showed a significant increase in micronuclei above control observed at the lowest dose of 0.0001 Gy, with a further increase at the higher dose of 0.001 Gy, above which the response plateaus up to 0.5 Gy, with evidence for a further increase at 1 Gy. Again, a very similar response is seen at the later time of 10 population doublings post irradiation, with a significant increase at all doses, with the response increasing from 0.0001 Gy to 0.001 Gy, above which the response stays pretty constant. A similar trend was observed after 20 population doublings; however, there was more dose-to-dose variability in response; a significant increase was only observed for 0.001 Gy, 0.01 Gy, 0.5 Gy and 1 Gy, but in part this may have been impacted by the rise in the control level of micronuclei observed at these later times.

The significant enhanced yield DNA damage detected by the comet assay at 0.0001 Gy, along with the corresponding significantly enhanced micronuclei yield is particularly surprising as only 0.1% of the cell nuclei and 0.8% of the cells will be traversed by alpha-particle tracks (Table 1). Based on the comet assay, the DNA damage induced appears to be greater when 0.1% of nuclei are traversed compared to that observed when 100% of the nuclei are traversed. Likewise, for micronuclei induction, the enhance response plateaus as when 1% of nuclei and 8% of cells are traversed with a similar response observed as the frequency of traversals are increased to 100% of nuclei and cells.

A significantly enhanced yield of micronuclei above controls (3-fold) was also observed following low doses of alpha-particles between two confluent AG1522 normal human-diploid skin fibroblasts [41]. This 3-fold enhancement was observed at doses between 0.01 to 0.003 Gy, but only a 4-fold increase after 0.1 Gy, even though ~10% more cells will be traversed. It was also found that at the lowest dose, the presence of lindane (inhibitor of gap junction-mediated intercellular communication) significantly reduced the observed enhancement in micronuclei supporting the occurrence of DNA damage in non-hit bystander cells. Likewise, Lin et al. [42] (2014) observed that the induction of sister chromatid exchanges in CHO and L-1 cells following an alpha-particle dose of 0.0014 Gy where only 0.8% of the nuclei were traversed. A recent study [43] showed that low fluence of ^241^Am alpha-particles (0.1 to 1 Gy) induced DNA damage (MN) in both directly irradiated and bystander cells of Human adult dermal fibroblast (HADF) and peripheral blood lymphocytes (PBL) cells. However, damage of bystander effect was higher (6-fold) in cells co-cultured with the α-irradiated cells than that of with 6 MV X-irradiated cells. The DNA damage (MN) in alpha-particle-irradiated cells persisted for 10 days (approximately 7–8 population doublings) in the lymphocyte cells and for 4 population doublings in HADF cells.

The alkaline comet assay predominantly detects DNA single-strand breaks along with alkali- label sites. While these can be produced directly by ionising radiation, many of these simple lesions will be repaired within the hour following irradiation. However, these can also be endogenously produced as a result of reactive oxygen species (ROS) and other reactive metabolites such as reactive nitrogen species (RNS), with each cell in the body having a background level of at least 50,000 endogenous lesions per day. A temporary or persistent change in ROS and/or RNS concentrations can result from the perturbation in homeostasis of signalling within the cells, resulting in a modulation of the background rate of endogenous DNA damage induction [44]. At the low doses used (≤1 Gy), it is likely that modulation of endogenous damage will dominate the response; however, at doses above 1 Gy (typically used in radiobiology experiments), it is expected that DNA damage induced directly by ionising radiation will increasingly dominate the response resulting in the expected increasing response with an increasing dose for both the comet assay and micronucleus induction at the early time point. At higher doses, the yield of micronuclei is expect to increase with the dose, as the micronucleus assay is a commonly used biodosimetry technique [42,45].

Previous experiments have clearly shown that high-LET alpha-particle irradiation of normal cells can perturb intercellular signalling even at very low doses associated with typical human exposures and elicit response in cells including non-hit bystander cells [43,44]. In these experiments, alpha-particles were observed to induce apoptosis in non-irradiated transformed 208Fsrc3 fibroblast cells following co-culture with irradiated normal 208F fibroblast cells at a dose as low as 0.00029 Gy, where only 1.1% of the cells were traversed. The apoptosis levels reached a plateau at 0.025 Gy, where 96% of cells were traversed by alpha-particles. A range of inhibitors were used to demonstrate theses effects, which resulted from the modulation of ROS/RNS (including OH, NO and peroxidase) and TGF-β signalling. Furthermore, TGF-β and ROS have been reported as mediators in the induction of bystander effects in normal human diploid lung fibroblasts (HFL1) following alpha-particle irradiation at 0.01–0.19 Gy [46].

In addition to the early induction of DNA damage, the results show an enhanced level of damage detected using both the comet and micronuclei assays at 10 and 20 population doublings following irradiation with low doses of alpha-particles. These responses are consistent with radiation-induced genomic instability (GI), where a persistent elevation of DNA damage can result in the production de novo non-clonal gene mutations and aberrations in the progeny of the irradiated cell populations [46,47,48,49]. The frequency of these mutations in the progeny typically occur at a higher frequency than would be expected than if they were the result of an mutator mutation, with the response typically plateauing at low doses. Genomic instability is a well-recognised feature of many tumours with its ability to generate genetic diversity [12]. Pioneering experiments of [14,50] showed a significant induction of radiation induced GI in progeny of hemopoietic stem cells following irradiation with alpha-particles at doses from 0.25 Gy–1 Gy. Subsequent analysis demonstrated that the instability phenotype was not only observed in cells that had been traversed by an alpha-particle but also non-hit bystander cells as a result of intercellular signalling [13,27,51,52]. Subsequent studies also showed that GI in HF19 normal human fibroblast following exposure to 0.4 Gy alpha-particle at both early (at 3 population doublings following irradiation) and delayed (at 20 and 35 population doubling) time points compared to control cultures [53]. Additionally, Ghandhi et al. [54] (2008) found that both direct and bystander exposure of human fibroblast IMR-90 lung cells at 0.5 Gy alpha irradiation elevated micronucleus induction and modulated the gene expression at 24 h following irradiation.

In addition to ROS and RNS signalling, exosomes are known to have an important role in intercellular signalling and have been implicated in causing epigenetic changes, potentially as a result of radiation induced histone modifications [55]. A previous study showed that exosomes and their cargo (proteins and RNA) can communicate radiation-induced bystander effects following the irradiation of MCF7 breast epithelial cancer cells with 2 Gy X-rays with a reduction of telomerase activity in both the irradiated and bystander cells [28]. In this study a significant increase in exosome concentration was observed 24 h post exposure to 1.5 Gy of X-rays over non-irradiated controls, while there is a suggestion of a small decrease in concentration following exposure to 0.4 Gy alpha-particles (Figure 5). However, the observed control level observed for the alpha-particle study was also high, so it is possible that the concentration may also be sensitive to the procedure used. While a large increase in the DNA in the comet tail was observed in cells irradiated with 1.5 Gy X-rays assayed 48 h post exposure, no significant increase was observed in non-irradiated cells exposed to exosomes from X-ray irradiated cells (exosomes transferred 24 h post exposure). Interestingly, a significant bystander response (percentage of DNA in comet tail) was observed in non-irradiated cells exposed to exosomes from both 0.001 Gy and 0.4 Gy alpha-particle irradiated cells. The response observed in the 0.001 Gy alpha-particle cell population (where 1% of the nuclei are traversed) also appears to be higher than that observed for the higher dose of 0.4 Gy (98% of nuclei traversed). Thus, this indicates the potential role of exosome-mediated DNA damage effects in bystander cells following alpha-particle irradiation. However, we still have to elucidate if these can also produce effects in the progeny of the exposed cells.

## 5. Conclusions

In conclusion, alpha-particle irradiation at low doses from 0.0001–1 Gy was observed to produce a significant induction of micronuclei (at 5 h following irradiation) and DNA damage (at 1 h following irradiation) in HF19 cells shortly after irradiation. Interestingly, this damage was still evident and statistically significant in all irradiated groups until 10 population doublings. At 20 population doublings, similar trends were observed, but the response was a little more variable with only a significant increase observed at 0.0001 Gy and 0.01 Gy for the comet assay and 0.001 Gy, 0.01 Gy 0.5 Gy and 1 Gy for the induction of micronuclei in binucleate cells. These results are consistent with the induction of GI across all the alpha-particle doses used in not only the irradiated cells, but also the significant number of non-irradiated cells present in the cell population exposed at the lower doses used. While it is likely that a persistent increase in the endogenous damage levels due to the perturbation of ROS/RNS signalling and associated oxidative stress may be a factor, the data also indicated that effects can be mediated via exosomes, which have the potential to expand the physical range of these effects.

The induction of GI in the progeny of cells exposed to very low doses of alpha-particles, even those not actually traversed, may have important implications when evaluating the cancer risk associated with alpha-particle exposure. Additionally, the analysis of exosomes released following alpha-particle exposure shows that their impact in biological response following irradiation should be explore further and may also prove an interesting area of research in the search to find useful biomarkers of exposure.

## Figures and Tables

**Table 1:** The variation in the average number of alpha-particle track traversals per cell and per nucleus for the HF19 cell line and corresponding calculated percentages of nuclei and cells traversed as a function of the alpha-particle dose. The calculations were based on average cellular and nuclear areas of 1557 μm^2^ and 189 μm^2^, respectively.

Dose (Gy)	Calculated Average α-Particle Track Traversals per Cell (% of Cells Traversed by 1 or More Tracks)	Calculated Average α-Particle Track Traversals per Nucleus (% of Nuclei Traversed by 1 or More Tracks)
0.0001	0.008 (0.80%)	0.00098 (0.098%)
0.001	0.080 (7.73%)	0.0098 (0.967%)
0.01	0.804 (55.3%)	0.0976 (9.3%)
0.1	8.04 (100%)	0.976 (62.3%)
0.5	40.2 (100%)	4.88 (99.2%)
1	80.4 (100%)	9.76 (100%)

## Supplementary Materials

The following are available online at https://www.mdpi.com/2079-7737/10/1/11/s1, Figure S1: Percentage of viable HF19 cells at 48 h direct and exsosome bystander alpha particle irradiation, Figure S2: Percentage of viable HF19 cells at 48 h direct and exsosome bystander X-ray irradiation, Figure S3: Florescent microscope images for HF19 cells subjected to comet assay at 48 h following direct X-ray and alpha particle irradiation and 24 h following exosome bystander transferred to non-irradiated cells.
