# Supplementary material for: Induction of Genomic Instability in a Primary Human Fibroblast Cell Line Following Low-Dose Alpha-Particle Exposure and the Potential Role of Exosomes
Source: Biology (Basel). 2020 Dec 28;10(1):11. doi: 10.3390/biology10010011 (PMC7824692; doi:10.3390/biology10010011)
Supplement: Supplementary file 1 [file biology-10-00011-s001.pdf]

biology

MDPI

Supplementary Materials

Induction of Genomic Instability in A Primary Human Fibroblast Cell Line Following Low-Dose Alpha-Particle Exposure and the Potential Role of Exosomes

Eman Mohammed Elbakrawy, Ammar Mayah, Mark A. Hill and Munira Kadhim

Cell viability

The percentages of both dead and viable cells following irradiation were measured using the Cell Analyser (Muse) according to the DNA-binding dyes' permeability to the reagent, i.e. dead cells lose their membrane integrity, allowing the dye to stain their nuclei, thus allowing differentiation from the non-stained live cells (Laka et al., 2019) https://www.mdpi.com/2073-4425/10/1/41). The viability assay was performed at 48 hours following irradiation.

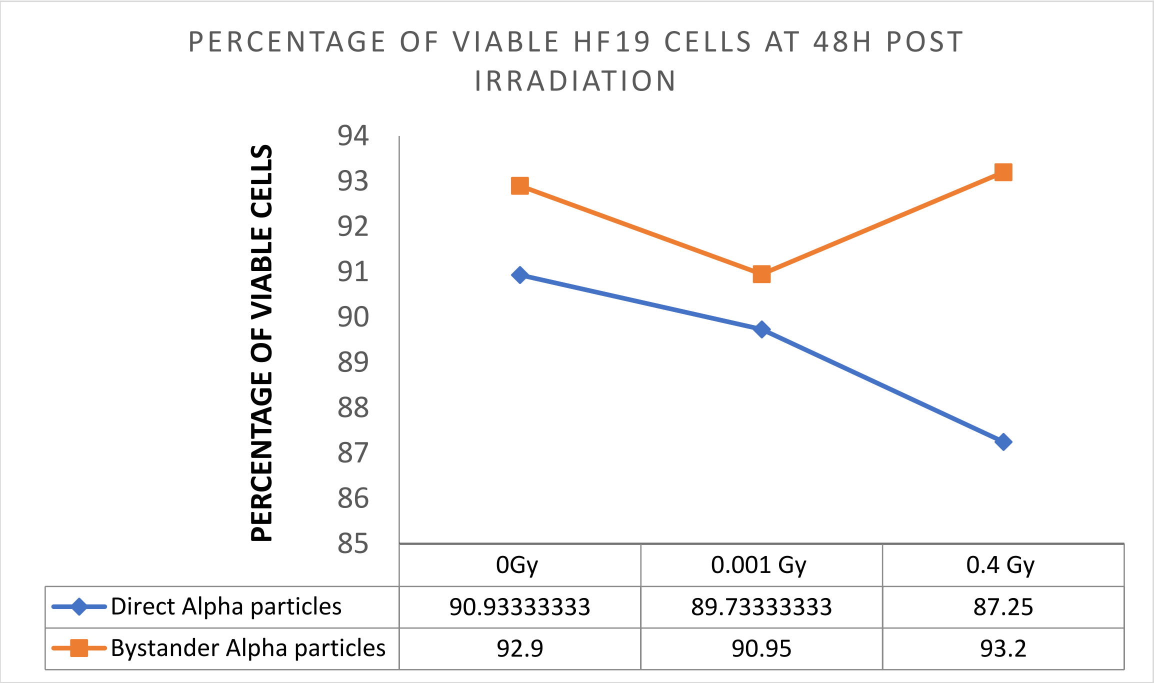

	0Gy	0.001 Gy	0.4 Gy
Direct Alpha particles	90.93333333	89.73333333	87.25
Bystander Alpha particles	92.9	90.95	93.2

Figure S1. Percentage of viable HF19 cells at 48 h direct and exsosome bystander alpha particle irradiation. The data showed a reduction in the percentage of viability in the direct irradiated groups (0.001 and 0.4 Gy) compared to the corresponding control. This decrease in cell viability was statistically insignificant. The exosome bystander data showed a slight decrease in the percentage of viability in HF19 cells at 24 h following transferring irradiated exosomes from 0.001 Gy irradiated cells to non-irradiated cells. The data for the exosome bystander 0.4 Gy irradiated group showed a slight increase in the percentage of viability compared to corresponding control.

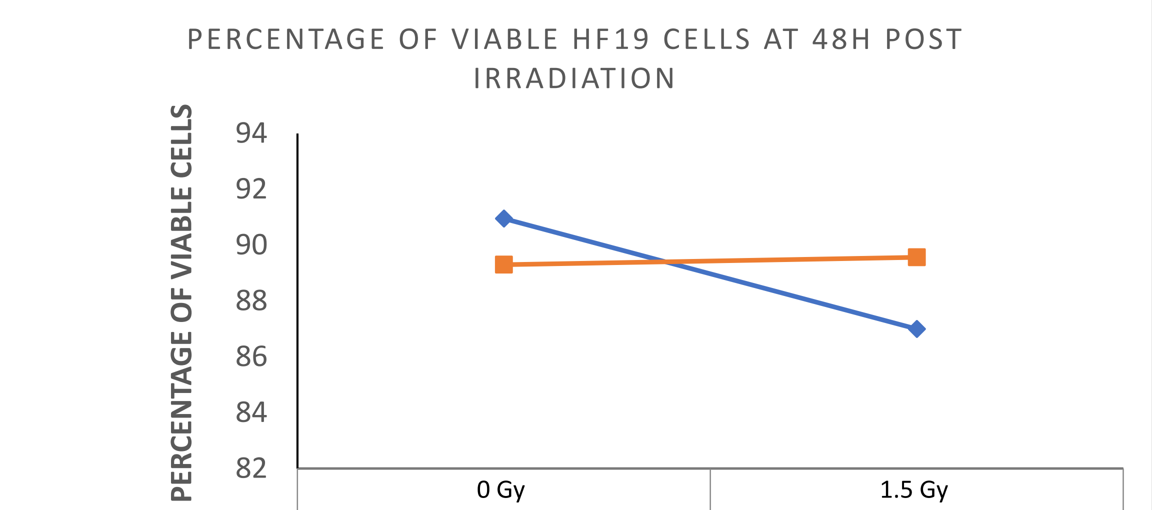

	0 Gy	1.5 Gy
Direct x-ray	90.95	87
Bystander x-ray	89.3	89.56666667

Figure S2. Percentage of viable HF19 cells at 48 h direct and exsosome bystander x-ray irradiation. The data showed the percentage of viability was lower in 1.5 Gy direct irradiated cells compared to the corresponding control. This decrease in cell viability was not statistically significant. The exosome bystander data showed a slight increase in the percentage of viability in HF19 cells at 24 h following transferring irradiated exosomes to non-irradiated cells.

Alpha-particle

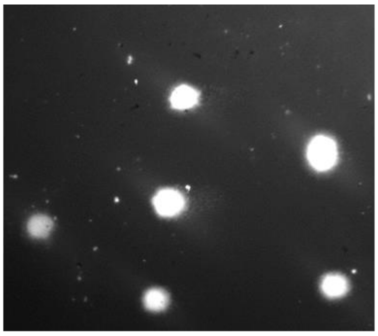

0Gy, alpha particles

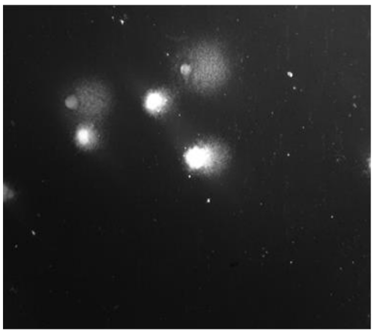

0.001 Gy, Direct alpha particles

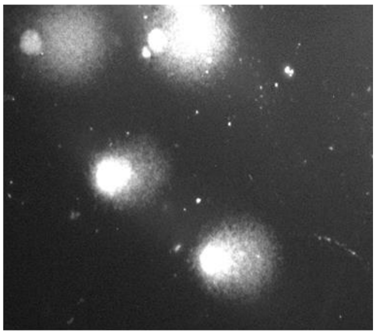

0.4 Gy, Direct alpha particles

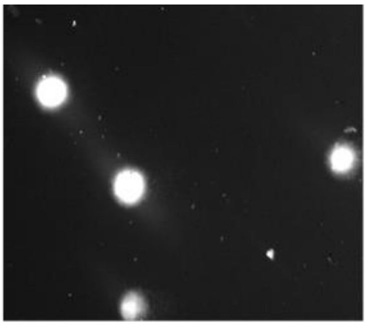

0Gy exosome Bystander, alpha particles

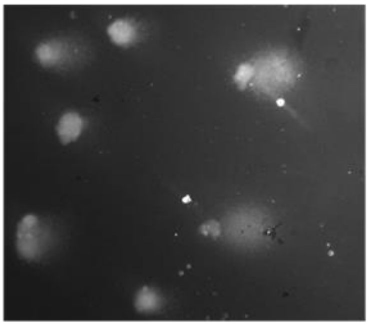

0.001 Gy, Exosome bystander alpha particles

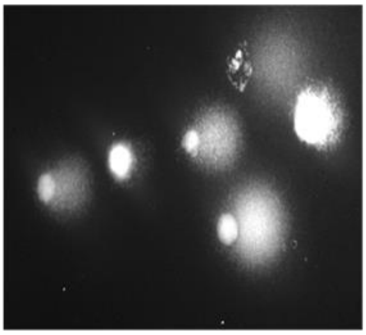

0.4 Gy, Exosome bystander, alpha particles

X-ray

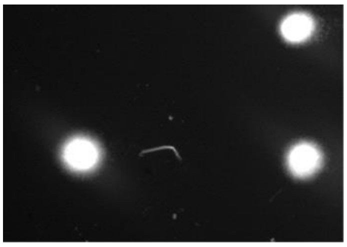

0Gy, x ray

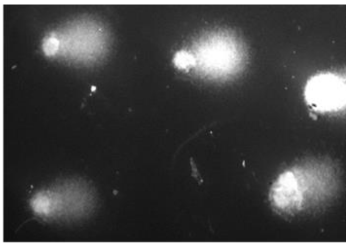

1.5Gy x-ray, Direct

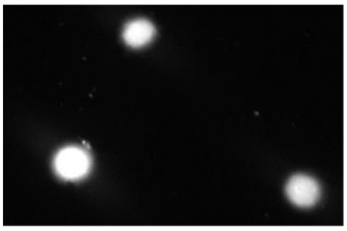

0Gy Bystander exosome, x ray

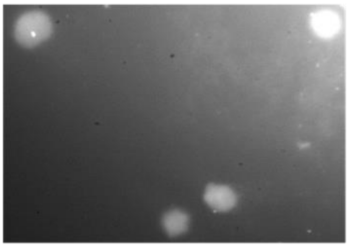

1.5Gy exosome Bystander, x-ray

Figure S3. Florescent microscope images for HF19 cells subjected to comet assay at 48 h following direct x-ray and alpha particle irradiation and 24 h following exosome bystander transferred to non-irradiated cells.
